# Acute Spontaneous Coronary Artery Thrombosis as Initial Presentation of HIV Infection in a Young Man

**DOI:** 10.1155/2015/342348

**Published:** 2015-03-02

**Authors:** James Kayima, Wilson Nyakoojo, Damalie Nakanjako, Marco A. Costa, Christopher T. Longenecker, Daniel I. Simon

**Affiliations:** ^1^Department of Medicine, School of Medicine, College of Health Sciences, Makerere University, P.O. Box 7072, Kampala, Uganda; ^2^Uganda Heart Institute, Ward 1C, Mulago Hospital Complex, P.O. Box 7051, Kampala, Uganda; ^3^Infectious Disease Institute, Makerere University, P.O. Box 22418, Kampala, Uganda; ^4^Harrington Heart and Vascular Institute, University Hospitals Case Medical Center, Case Western Reserve University, School of Medicine, 11100 Euclid Avenue, Cleveland, OH 44106, USA

## Abstract

*Introduction*. The presentation of acute coronary syndrome (ACS) in young HIV patients may be atypical with different pathophysiological and clinical features. Acute coronary thrombosis, as a presentation of acute coronary syndrome in young patients with HIV, raises diagnostic and treatment challenges. *Case Presentation*. We describe a case of a 33-year-old African man, without traditional atherosclerotic risk factors, who presented with chest pain of acute onset. Emergent coronary angiography revealed extensive thrombus in the left anterior descending coronary artery with no evidence of atherosclerosis in this or other coronary vessels. Plaque and/or thrombus prolapse through the stent was noted following percutaneous coronary intervention (PCI). Resolution of chest pain and improvement in ST-segment elevation was noted after the procedure. A diagnosis of HIV infection was made during the workup for HIV infection. *Conclusion*. In young patients without traditional risk factors, HIV infection is a possible etiological factor for spontaneous coronary artery thrombosis. Percutaneous coronary intervention in patients with this presentation may be compounded with atherothrombotic complications. The likely pathophysiological pathway is superficial endothelial cell denudation as a result of chronic inflammation and immune activation.

## 1. Introduction

An increased risk of myocardial infarction has been documented in HIV-infected patients since the advent of efficacious antiretroviral therapy (ART) that prolongs life. An increased prevalence of traditional risk factors (e.g., smoking), uncontrolled viral replication, and metabolic toxicities of ART have been proposed as underlying drivers of atherosclerosis and risk for ACS [1], yet few studies have been conducted in sub-Saharan Africa [2]. Spontaneous coronary thrombosis in young HIV individuals has been described in patients on antiretroviral therapy [3, 4]. We report a somewhat rare case of a 33-year-old man with previously undiagnosed HIV-infection who emergently presented with an anteroseptal ST-segment-elevation myocardial infarction (STEMI). Coronary angiograms revealed extensive thrombus in the left anterior descending artery with minimal angiographic coronary artery disease and no spontaneous coronary artery dissection. This presentation may pose challenges in management.

## 2. Case Presentation

**Figure 1:**
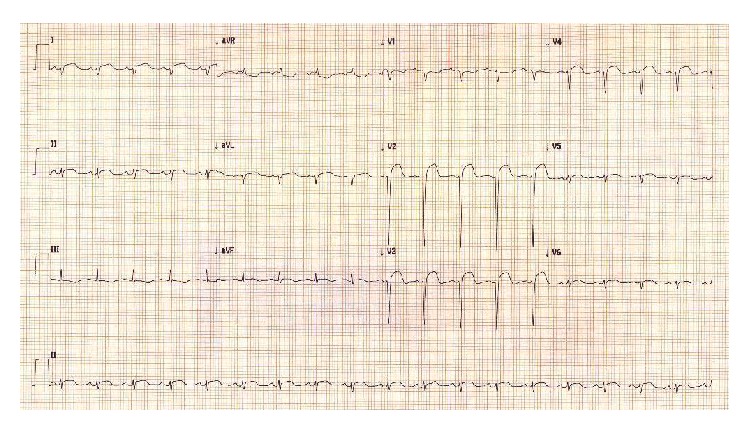
ECG on admission showing anterolateral ST elevation.

A 33-year-old man was referred urgently to our medical facility. He reported a history of intermittent chest pain over the preceding two weeks. He attributed the chest pain to musculoskeletal issues due to lack of physical activity, and he had resorted to aerobic exercise sessions for relief. Two hours before presentation to the referring medical unit, he developed severe retrosternal chest pressure accompanied by dyspnea. An ECG performed at the referring hospital revealed ST-segment elevation V1–V6 consistent with an anterolateral STEMI and he was promptly transferred to our cardiac care unit (Figure 1).

His past medical history was negative for hypertension, diabetes, dyslipidemia, or smoking. He had no family history of cardiac problems or sudden death. Social history was negative for recreational drug use.

Physical examination revealed an overweight young man (BMI = 27) who was uncomfortable secondary to chest pain. He was afebrile. Blood pressure was 141/81 mmHg and equal in arms, heart rate 62 beats/min, and respiratory rate 16 breaths/min. The chest was clear to auscultation. He had no jugular venous distention and cardiac auscultation revealed normal S1 and S2 with no S3 or murmurs.

Because the patient had persistent chest pain and ST-segment-elevation, a decision was made to take him for urgent left heart catheterization, coronary angiography, and primary PCI. The patient was pretreated with aspirin 325 mg, prasugrel 60 mg oral load, and unfractionated heparin 4,000 units IV bolus. During preparation for the procedure, the initial laboratory data returned. These revealed creatinine of 74 *μ*g/mL (unknown baseline), a normal complete blood count (CBC), and coagulation study. Liver function tests were abnormal for alkaline phosphatase of 154 (35–105), alanine aminotransferase (ALT) 39 (7–35), and aspartate aminotransferase (AST) 72 (13–35). An initial set of cardiac enzymes were mildly elevated with a creatine kinase myocardial band (CKMB) mass of 13.00 ng/mL (normal <4) and troponin of 0.1 ng/mL (normal 0.0 to 0.04). His lipid panel was within normal limits, and a urinalysis showed proteinuria. Screening for infection, which is routine at our facility, revealed normal hepatitis B and C serology. The HIV antibody test was positive. His CD4+ count was 643 cells/*μ*L.

**Figure 2:**
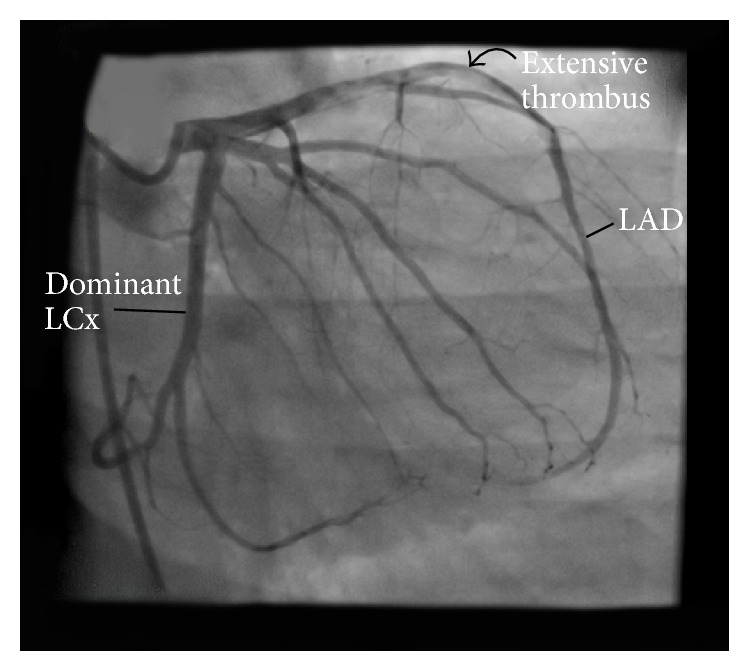
Preintervention angiographic image showing extensive LAD thrombosis. LAD: left anterior descending coronary artery; LCx: left circumflex coronary artery.

**Figure 3:**
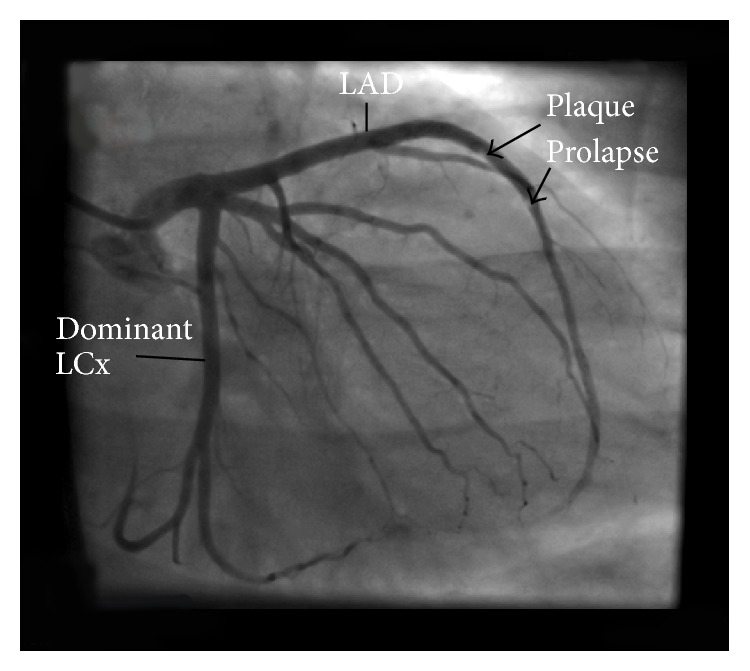
Postintervention angiographic image showing plaque prolapse. LAD: left anterior descending coronary artery; LCx: left circumflex coronary artery.

Coronary angiography revealed extensive, nonocclusive thrombus extending from proximal to mid left anterior descending (LAD) coronary artery with no significant atherosclerosis in this or other vessels (Figure 2). A decision was made to intervene on the LAD lesion. The left coronary artery was engaged with 6F EBU 3.5 guide catheter. A 0.014 Asahi Prowater guide wire was used to cross the thrombotic lesion and advanced into the distal LAD. Three thrombectomy passes were made with an Export catheter (Medtronic, Minneapolis, Minnesota, USA) yielding moderate amount of red (i.e., red blood cell-rich) and white (i.e., platelet-rich) thrombi. Follow-up angiography revealed large residual thrombus burden. Two Resolute Integrity (Medtronic) drug eluting stents (3.5 × 26 and 3.0 × 30) were deployed in an overlapping fashion to cover the thrombotic lesion. The stents were postdilated with a 3.5 × 20 noncompliant balloon to 18–22 atmosphere of pressure. Follow-up angiography after intracoronary nitroglycerin revealed TIMI II flow and mild plaque and/or thrombus prolapse through the distal stent (Figure 3). At the conclusion of the case, the patient was pain free with near ST-segment resolution.

The patient was then admitted to the coronary care unit for monitoring and post-PCI care. Echocardiography revealed overall preserved left ventricular function with anterior hypokinetic and left ventricular ejection fraction of 55%. Treatment with dual antiplatelet therapy (aspirin 81 mg and prasugrel 10 mg), enalapril, and rosuvastatin was instituted. He was counselled regarding his HIV status and the infectious disease team was engaged to make arrangements for outpatient HIV care.

## 3. Discussion

We report an uncommon case of a patient previously unaware of his HIV status, who presented with STEMI secondary to large thrombus burden in the LAD. HIV infection is thought to be a risk factor for MI with an adjusted hazard ratio of 1.5–2 [5]. A higher prevalence of traditional coronary artery disease risk factors in HIV, viral specific factors, and the toxicity of ART have been proposed as pathophysiologic factors promoting atherosclerosis in HIV populations [6]. As a result, MI may occur at younger ages among HIV-infected compared to uninfected populations.

While premature atherosclerosis has been reported in most HIV cases with ACS, there have been very few reported cases of young HIV patients with coronary artery thrombosis with no significant angiographic coronary artery disease. Previously documented cases are notable for known HIV infection with concomitant antiretroviral treatment [3, 4]. In our patient, whose diagnosis of HIV infection was made in the STEMI setting, the duration of HIV infection could not be ascertained.

The differential diagnosis for MI in the absence of atherosclerosis includes, among others, coronary artery dissection, superficial endothelial cell erosion (typically associated with younger age, female gender, and diabetes), and coronary artery embolism from left atrial appendage or mural thrombus, endocarditis, and paradoxical fat emboli [7]. In this case, there was no evidence for coronary artery dissection or embolism from subclinical endocarditis or previously unrecognized intracardiac thrombus. To the best of our knowledge, there is no clear association between HIV infection and superficial endothelial cell erosion, which is an angioscopic or pathologic diagnosis.

HIV-associated chronic inflammation and immune activation have been shown to be potential risk factors for cardiovascular disease [8]. The resultant endothelial dysfunction, typically assessed as defective endothelial-dependent vasodilatation of the brachial artery, may be the mechanistic link between HIV infection and acute atherothrombotic events. In this setting, apoptosis of endothelial cells may contribute to desquamation of endothelial cells in areas of superficial erosion [9]. Recent data support the notion that HIV infection itself rather than antiretroviral therapy induces endothelial dysfunction with both micro- and macrovascular abnormalities correlating with CD4 counts in various studies [10]. However, data associating low CD4+ T-cell counts with major adverse cardiovascular events is mixed [11]. In the present case, the patient was antiretroviral therapy naïve, albeit with a relatively spared immune system as evidenced by a high CD4 count of 643 cells/*μ*L.

ACS due to extensive coronary artery thrombus confers significant treatment challenges. Slow flow and no-reflow secondary to distal embolization are associated with periprocedural infarction and increased mortality. We employed aspiration thrombectomy and direct deployment of a stent to minimize distal embolization in our patient, but it is noteworthy that final angiographic flow was not normal (i.e., TIMI 2 grade flow). The direct stenting technique has been shown to reduce distal embolization by avoiding thrombus disintegration during predilation and using the stent as a scaffolding device to entrap thrombus [12]. When plaque and/or thrombus prolapse occurs, it is controversial whether additional balloon inflations and stent deployment are required. Intravascular imaging-guided studies suggest that endogenous thrombus dissolution typically occurs with no increase in stent thrombosis rates or neointimal formation compared to those without thrombus or plaque prolapse [13].

## 4. Conclusion

This case illustrates an ACS caused by spontaneous LAD thrombosis in a patient with HIV infection. It is likely that the pathogenesis for this event was related to increased risk of thrombosis associated with inflammation, immune activation, and thrombophilia as a result of uncontrolled HIV infection. It is also intriguing to speculate whether this patient experienced superficial endothelial cell erosion as a consequence of HIV infection. Management of the patient was challenging due to high thrombus burden. As primary PCI becomes a viable reperfusion option for STEMI in sub-Saharan Africa, there is a unique opportunity to further investigate the mechanisms of ACS in the HIV-infected population.
